# BuscoPhylo: a webserver for Busco-based phylogenomic analysis for non-specialists

**DOI:** 10.1038/s41598-022-22461-0

**Published:** 2022-10-17

**Authors:** Alae-Eddine Sahbou, Driss Iraqi, Rachid Mentag, Slimane Khayi

**Affiliations:** Biotechnology Research Unit, Regional Center of Agricultural Research of Rabat, National Institute of Agricultural Research, Avenue Ennasr, Rabat Principale, BP 415, 10090 Rabat, Morocco

**Keywords:** Phylogeny, Software

## Abstract

Here we present the BuscoPhylo tool that enables both students and established scientists to easily perform Busco-based phylogenomic analysis starting from a set of genomes sequences. BuscoPhylo is an efficient and user-friendly web server freely accessible at https://buscophylo.inra.org.ma/. The source code, along with documentation, is freely available under an MIT license at https://github.com/alaesahbou/BuscoPhylo.

## Introduction

In the last two decades, the development of next generation sequencing technologies of DNA revolutionized the way of deciphering the genome patrimony of living organisms. As results, the cost of DNA sequencing decreased drastically leading to an unprecedented flood of genomic data that is generated through diverse sequencing platforms around the world. This makes the development of high-throughput approaches for handling storage, management and analysis of huge volumes of data, ever more important to overcome the bottleneck in biological discovery.

Phylogenetic analyses lie at the core of the genomics analysis methods leading to the reconstruction of the evolutionary history of organisms^1^. Thus, taking advantage of the huge amounts of sequencing data available for both model and non-model organisms, the traditional molecular phylogenetics approaches have transformed into phylogenomics where genome-scale data is integrated^2^. As a consequence, this approach leads to insightful gains in terms of phylogenetically informative characters compared to the few loci used in traditional phylogenetic studies that could be hampered by frequent horizontal gene transfer events, or the low phylogenetic signal of traditional markers (housekeeping genes, SSU, etc.)^3,4^. Through the use of multiple loci, the phylogenomic approaches provide concrete description of molecular evolution and highly resolved relationships between groups and taxa on the tree of life^1,5–7^. To construct a phylogenetic relationship, the tree estimation should be based on orthologous loci whose common ancestor diverged as result of speciation, thus the resulting phylogenetic tree will be congruent with the species tree^4^. While the number and the nature (nucleotide or proteins) of considered loci in the analysis will undoubtedly influence the result of the phylogenomic analysis, defining the suitable locus for the phylogeny inference is therefore a crucial step in these approaches^4^. Identification of orthologous genes between a set of genome sequences is not an obvious task and may be burdened by the choice of orthology prediction methods^4,8,9^. The OrthoDB database of orthologues (www.orthodb.org) constitutes a hierarchical catalog of animal, fungal and bacterial orthologous genes based on pairwise sequence comparisons^10^. This is used to compile the Benchmarking Universal Single-Copy Orthologs (BUSCO) set for 193 lineages (67 eukaryotic, 83 bacterial, 16 archaeal, and 27 viral datasets) of living organisms using similarity-based methods^11–13^. The BUSCO sets are used to provide quantitative measures of the completeness and quality of genome, transcriptome assemblies as well as annotated gene sets. In addition, being near-universal single-copy genes, BUSCO are suitable markers for integration in phylogenomic studies^12^.

Technically, phylogenomic studies often include complex pipelines involving many steps and several tools and scripts, such as downloading, renaming, reformatting the sequences, identification of homologous sequences, alignments and graphical rendering; this makes it challenging for scientists lacking programming experience and willingness to harness novel methods and data. Many user-friendly programs and web sites were developed to provide phylogenetic analysis from sequences sets but none of them have addressed phylogenomic approaches^14–17^.

In this context, BuscoPhylo, is implemented to provide a fully automated and complete pipeline intending to quickly perform BUSCO-based phylogenomic analysis starting from genome assembly, annotated gene set, or transcriptome assembly as single input and the taxonomic domain of origin (bacteria, archaea, or eukaryota). BuscoPhylo is a free, on-line and user-friendly webserver that enables the user to export phylogenomic trees ready for use in publication.

## Methods

BuscoPhylo was developed with PHP, Python and Bash scripts as backend and configured on an Ubuntu Linux operating system with an Apache server (http://www.apache.org/). HTML5, CSS3 and JavaScript were used as the front-end programming languages. The storage of the inputs uses MySQL. The hardware specification used to deploy BuscoPhylo has 32 Intel(R) Xeon(R) CPU (Central Processing Unit) E5-2699 v3 @ 2.30 GHz with 256 gigabytes in Random Access Memory (RAM). The storage space is composed of 4 disks each with 2 Terabytes. The specifications could be extended depending on the usage.

**Figure 1:**
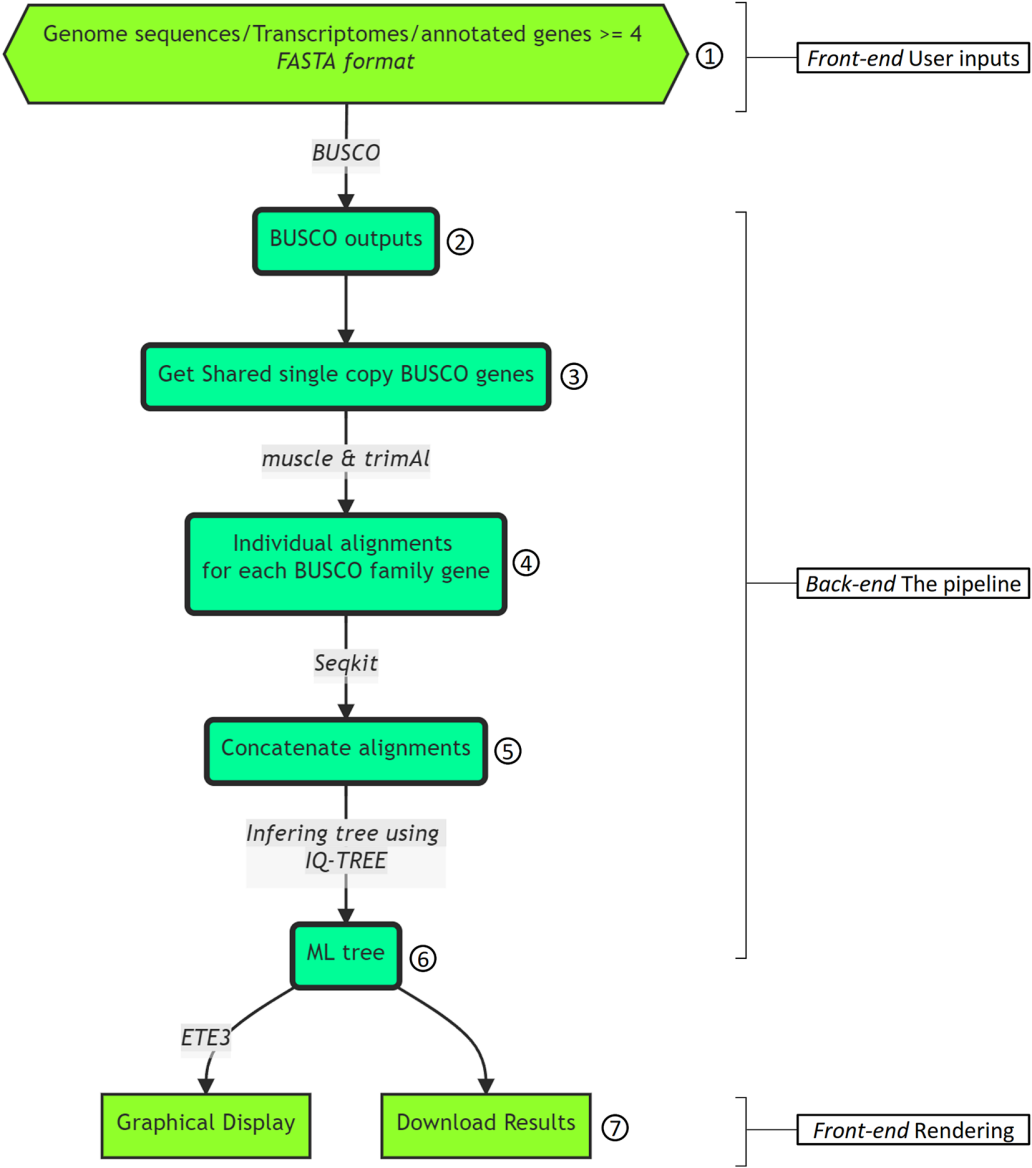
The BuscoPhylo pipeline steps; (1) In case of genomes as input sequence type, separate Genome sequence files are provided as inputs. (2) BUSCO searches are launched on each genome sequence with defined lineage. (3–5) The shared Busco Single Copy genes (S-BUSCO) are extracted, and individual alignments are performed for each BUSCO family gene before generating the super-matrix alignment. (6) the ML phylogenomic tree is inferred. (7) Finally, the graphical visualisation of the tree is displayed in addition to providing the overall results files.

BuscoPhylo receives FASTA files from the user based on the input sequence type (genome assembly, annotated gene set, or transcriptome assembly) and the taxonomic domain of origin (bacteria, archaea, or eukaryota) (Fig. 1) with a minimum of 4 input sequences. Once a project is created, the pipeline proceeds to run the Busco software^11–13^ for orthology prediction in each one of input sequences using the selected lineage and mode. Busco identifies the single-copy markers within input sequences in a runtime proportional to the size of BUSCO set (and eventually their domain) used and the sizes of input sequences. The runtime on the genome sequence type inputs generally is longer compared to transcriptome and annotated genes due to blastn searches^11^. The next step consists of retrieving the shared BUSCO (S-BUCSO) between queried input sequences and creating a multi-FASTA file for each BUSCO gene family using bash scripts. Then, individual alignments of protein sequences are performed using Muscle^18^. The runtime of alignment step depends on the number of genes and their length. Subsequently, the alignments are trimmed using trimAl^19^ to remove poorly aligned regions then concatenated into one super-matrix alignment using the Seqkit tool^20^. Afterwards, the multiple sequence alignment generated is used to infer the maximum likelihood (ML) tree using IQ-TREE^21^. By default, IQ-TREE determines the best-fit substitution model using ModelFinder^22^ followed by tree reconstruction. The generated tree file is visualized using ETE Toolkit^23^ for a quick assessment of the result. Finally, the user can download the entire result of the BuscoPhylo pipeline including the phylogenetic tree inferred in NEWICK*,* PNG, SVG and PDF formats for further analyses.

## Results and discussion

### Interface

**Figure 2:**
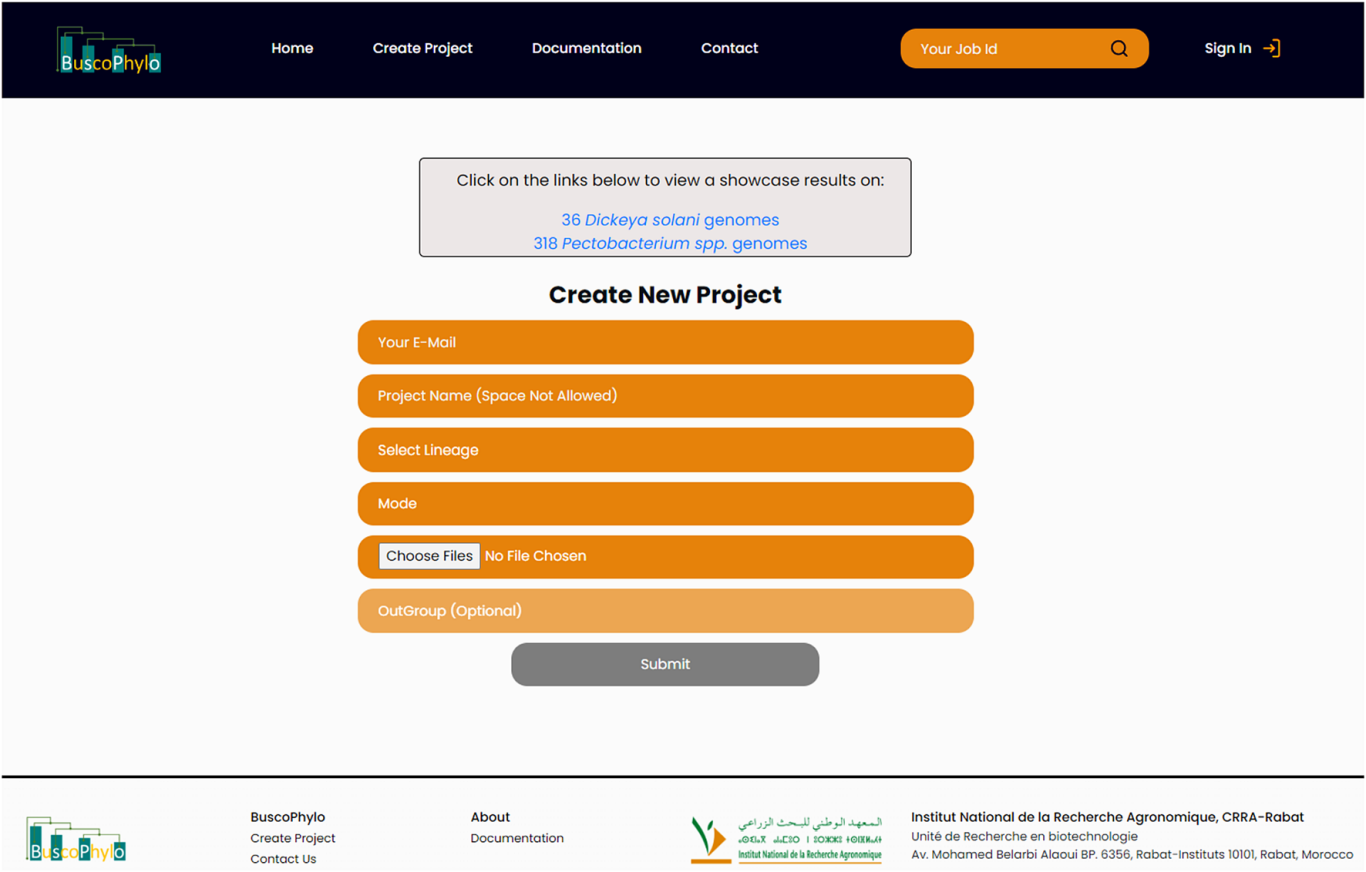
The BuscoPhylo interface for submitting a new phylogenomic project.

BuscoPhylo was developed with a Graphical User Interface (GUI) displayable in any modern internet browser without installation of any tool, or software. The interface has 5 required fields including the user e-mail address, project name, the lineage and the mode for Busco searches. An optional field is provided in case the user wants to root the phylogenetic tree (Fig. 2). BuscoPhylo enables the user to sign up for a personal account to facilitate the management of project submissions. The project manager implementation helps to check the project’s history and progress, re-export data and avoids redundancy in running projects. To tackle issues of storage capacity, each project is stored for a period of 1 month.

The user provides query sequences in FASTA format, then chooses the appropriate mode and lineage for BUSCO predictions based on input sequence type (genome assembly, annotated gene set, or transcriptome assembly).

After project submission, BuscoPhylo redirect the user to a webpage containing information about the server load status and the project details as well as a URL allowing to the user to monitor the progress of the job. Additionally, an email is sent automatically to inform the user when the job is complete and where to access the results.

### Outputs and intermediates data

After the project is done, BuscoPhylo will display a webpage allowing to the user quickly check the phylogenomic tree generated and download the NEWICK source file that can be visualized using any external tool for customized display of the tree. Furthermore, the tool provides the ability to retrieve all intermediate files generated through the core pipeline including Busco output, S-BUSCO and their individual alignment, the super-matrix alignment as well as the log files generated during the process of tree inferring.

### Webserver and stand-alone version

We deployed BuscoPhylo online (at https://buscophylo.inra.org.ma/) which enables its use online without installation. Users can also install and run BuscoPhylo on their personal computers very easily after installing the required software. Advanced users can deploy BuscoPhylo on local or public servers to provide online access to other users. Advanced users can also contribute to the development of BuscoPhylo, as its source code is available in GitHub (https://github.com/alaesahbou/BuscoPhylo).

### Performance evaluation of BuscoPhylo

To avoid computation burden in the current BuscoPhylo hardware, we have restricted the number of inputs to 300 sequences for bacteria/archaea and to 25 sequences for eukaryotes with genome size equal or less than 80 Mbp and this is true for all modes of Busco searches (genome, transcriptomes, and proteins). The server can run two projects simultaneously allowing an efficient distribution of available CPU performance on the project to gain ratio in terms of runtime.

To assess the performance of BuscoPhylo, we submitted two datasets of genome sequences from two domains: prokaryotes and eukaryotes. The first set of sequences is composed of all 35 publicly available *Dickeya solani* genomes (as of 16 September 2022) obtained from NCBI with the removal of duplicated genomes (Supp. Table S1). *Dickeya dadantii* was used as an outgroup. The species has an average genome size equal to 4.9 Mbp. BuscoPhylo runtime was around 31 min 3 s, with CPU peak of 55.9% of CPU performance (Fig. S1).

The results show that the phylogenomic tree is constructed from 363 S-BUSCO genes within 36 species with a total length of 1181 31 amino acid position. The tree topology produced by BuscoPhylo is similar to that computed on concatenated core gene alignments on 22 *D. solani*^24^ strains pinpointing the existence of a divergent sub-clade of *D. solani* species^25^ (Supp. Fig. S1, URL: https://buscophylo.inra.org.ma/item/1660667670/Dickeya).

The second project is about the fungal pathogen *Fusarium oxysporum* species with genome size varying from 40 to 70 Mbp. The dataset contains 20 genome sequences from different *formae speciales* that were retrieved from NCBI in addition to *Fusarium gramineaum* as an outgroup (Supp. Table S1). The analysis on BuscoPhylo takes 16 h 55 min and 20 s and the CPU peak usage is also 56.6% of CPU performance. The phylogenomic tree was generated based on 3 409 BUSCO genes conserved in all 21 species with a total length of 1 991 966 amino acid positions. The phylogenomic tree produced highlights the 3 known major taxonomic clades within *Fusarium oxysporum* speices^26,27^. Furthermore, the topology shows that many *formae speciales* are clustered together, as previously shown by several reports (Supp. Fig S2, URL: https://buscophylo.inra.org.ma/item/1663056926/Fusarium)^28–30^.

### BuscoPhylo webserver scope

In summary, BuscoPhylo was designed to build a phylogenomic tree for the 3 domains of living organisms: bacteria/archeae, eukaryotes. The pipeline implements computationally-intensive steps including Busco predictions, alignments and tree inference, that need adapted infrastructure if we want to scale on big eukaryotic genome like plant and animal species. Although, the tool can be built in an enhanced local server to easily tackle eukaryotic sequences with more than 80 Mbp without limitation to the number of sequences. Actually, the web version of BuscoPhylo is more suited for prokaryotic genomes that can be handled within a very reasonable runtime, to assist technically, both established and non-specialist researchers in their phylogenomic studies.

## Conclusion

The phylogenomic approaches have evolved with the growing of genomic datasets tackling problems of incongruences and reconstructing a well-supported molecular revolutionary history of the studied organisms. The era of big data in genomics implies the development of straightforward GUI transformed pipelines and workflow to fix bioinformatics bottlenecks that hinder data analysis and interpretation. To our knowledge, BuscoPhylo is the first web tools developed for both non-specialists and specialists, which provides a complete automated phylogenomic pipeline starting from FASTA files to a ready for publication phylogenomic tree. This tool will fill the gap between two major pillars of genomics analysis methods that are genome assembly and phylogenomic analysis providing the researchers much more time to focus on interpretation and downstream analysis.

We are committed to maintain BuscoPhylo for at least 2 years, providing necessary upgrades in software components. In the future, we also intend on adding raw reads as starting point for prokaryotic organisms, and including an assembly step so users can go directly from post-sequencing step to phylogenomics analysis.

## Supplementary Information

Supplementary Information.

## Data Availability

All data and codes used to implement this tool are available through the webserver and Github repository respectively https://buscophylo.inra.org.ma/; https://github.com/alaesahbou/BuscoPhylo.
