# Supplementary material for: BuscoPhylo: a webserver for Busco-based phylogenomic analysis for non-specialists
Source: Sci Rep. 2022 Oct 17;12:17352. doi: 10.1038/s41598-022-22461-0 (PMC9576783; doi:10.1038/s41598-022-22461-0)
Supplement: Supplementary file 1 — Supplementary Information. [file 41598_2022_22461_MOESM1_ESM.pdf]

Supplementary Data: Supplementary Tables and Figures

BuscoPhylo: A webserver for Busco-based Phylogenomic Analysis for Non-specialists

Alae-Eddine Sahbo[1], Driss Iraqi, Rachid Mentag, Slimane KHAYI

Content

Supplementary Figures S1-S2
Supplementary Table S1

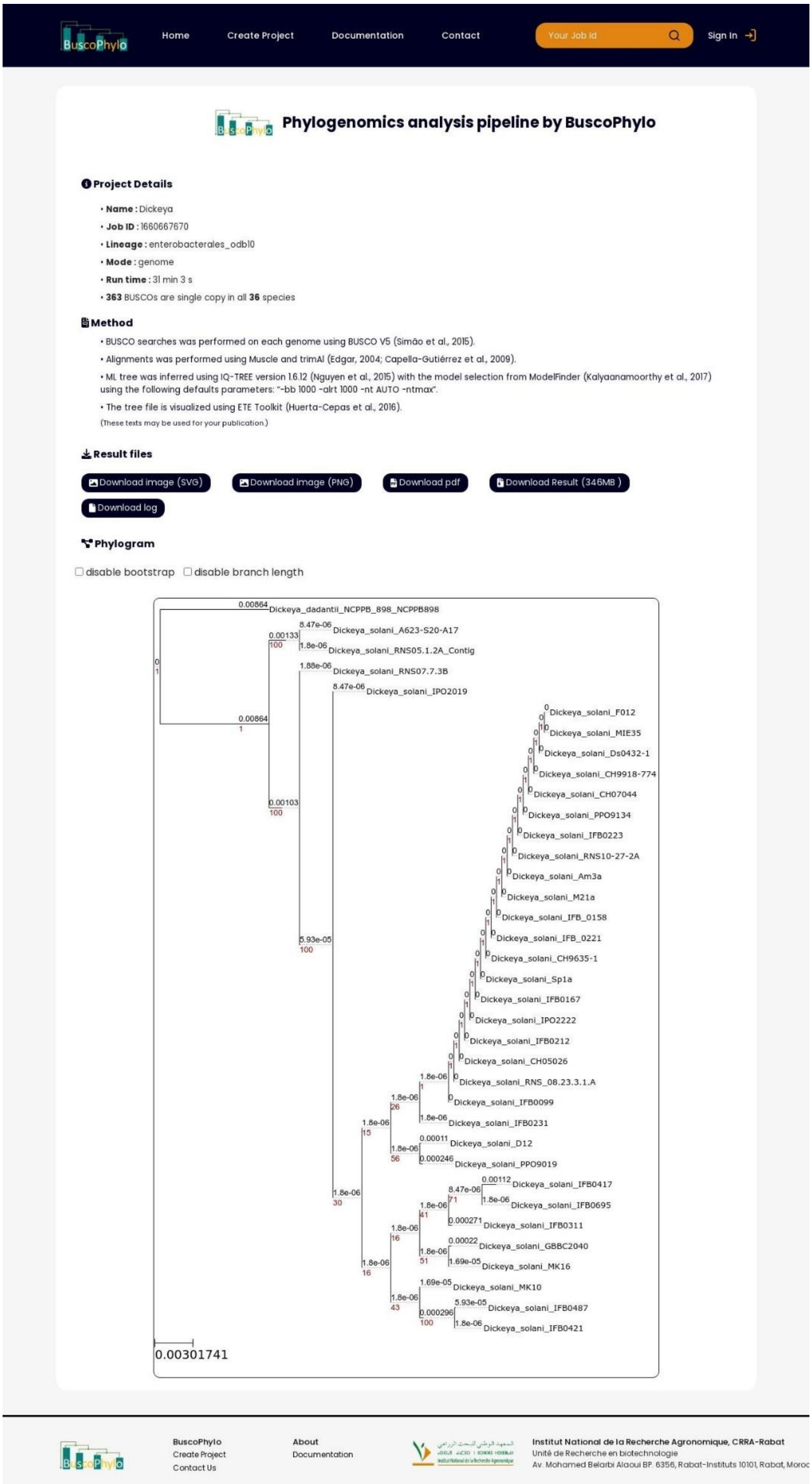

Supplementary Figure S1 BuscoPhylo output screenshot for *Dickeya solani* project. The entire result is accessible through the following URL: https://buscophylo.inra.org.ma/item/1660667670/Dickeya

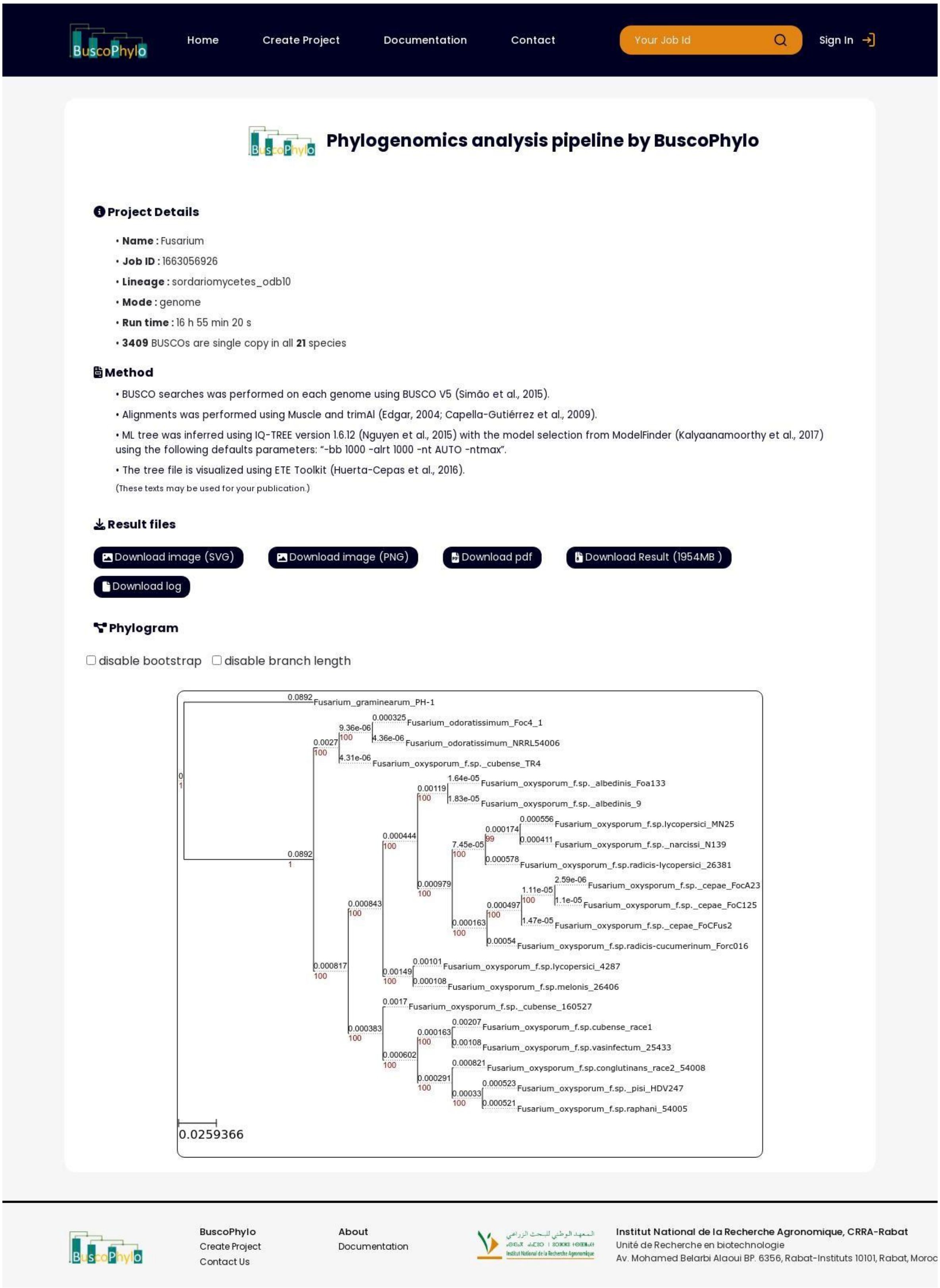

Supplementary Figure S2 BuscoPhylo output screenshot for *Fusarium oxysporum* project. The entire result is accessible through the following URL: https://buscophylo.inra.org.ma/item/1663056926/Fusarium

Supplementary Table S1 : The list of the organisms and their accession numbers used to asses BuscoPhylo performances

Domain	Species	Accession
Eukaryote	*Fusarium oxysporum* f. sp. *albedinis* 9	JAKELM010000000
	Fusarium oxysporum f. sp. *albedinis* Foa133	JAAVJG010000000
	Fusarium oxysporum f. sp. lycopersici 4287	AAXH01000000
	Fusarium oxysporum f. sp. lycopersici MN25	AGBH01000000
	Fusarium oxysporum f. sp. pisi HDV247	AGBI01000000
	Fusarium oxysporum f. sp. radicis-lycopersici 26381	AGNB01000000
	Fusarium oxysporum f. sp. vasinfectum 25433	AGNC01000000
	Fusarium oxysporum f. sp. cubense tropical race 4 54006	AGND01000000
	Fusarium oxysporum f. sp. conglutinans race 2 54008	AGNF01000000
	Fusarium oxysporum f. sp. raphani 54005	AGNG01000000
	Fusarium oxysporum f. sp. melonis 26406	AGNE01000000
	Fusarium oxysporum f. sp. cubense race 1	AMGP01000000
	Fusarium oxysporum f. sp. cubense race 4	AMGQ01000000
	Fusarium oxysporum f. sp. radicis-cucumerinum strain Forc016	MABQ02000000
	Fusarium oxysporum f. sp. cepae strain FoCA23	MRCW01000000
	Fusarium oxysporum f. sp. cepae strain FoCFus2	MRCU01000000
	Fusarium oxysporum f. sp. cepae strain FoC125	MRCV01000000
	Fusarium oxysporum f. sp. narcissi strain N139	MQTW01000000
	Fusarium oxysporum f. sp. cubense strain 160527	SRMI01000000
	Fusarium oxysporum f. sp. cubense strain TR4 isolate UK0001	VMNF01000000
	Fusarium graminearum PH-1	NZ_AACM02000000
Prokaryote	Dickeya dadantii NCPPB 898 NCPPB898	NZ_CM001976
	Dickeya solani A623-S20-A17	NZ_JAIZGA010000001
	Dickeya solani Am3a	NZ_VZQK01000001
	Dickeya solani CH05026	NZ_JAEKIO010000001
	Dickeya solani CH07044	NZ_JAEKIP010000001
	Dickeya solani CH9635-1	NZ_JAEKIM010000001
	Dickeya solani CH9918-774	NZ_JAEKIN010000001
	Dickeya solani D12	NZ_PGUT01000001
	Dickeya solani Ds0432-1	NZ_CP017453
	Dickeya solani F012	NZ_PDVN01000001
	Dickeya solani GBBC2040	NZ_CM001860
	Dickeya solani IFB0099	NZ_CP024711
	Dickeya solani IFB 0158	NZ_PENA01000001
	Dickeya solani IFB0167	NZ_CP051457
	Dickeya solani IFB0212	NZ_JABAON010000001
	Dickeya solani IFB 0221	NZ_PEMZ01000001
	Dickeya solani IFB0223	NZ_CP024710
	Dickeya solani IFB0231	NZ_CP051458
	Dickeya solani IFB0311	NZ_JABAOO010000001
	Dickeya solani IFB0417	CP051459
	Dickeya solani IFB0421	NZ_CP051460
	Dickeya solani IFB0487	JABAOP010000001
	Dickeya solani IFB0695	NZ_JABAOQ010000001
	Dickeya solani IPO2019	NZ_CP071062
	Dickeya solani IPO2222	NZ_CP015137
	Dickeya solani M21a	NZ_VZQM01000001
	Dickeya solani MIE35	NZ_VZQI01000001
	Dickeya solani MK10	NZ_CM001839
	Dickeya solani MK16	NZ_CM001842
	Dickeya solani PPO9019	NZ_CP017454
	Dickeya solani PPO9134	NZ_JWLT01000021
	Dickeya solani RNS05.1.2A Contig	NZ_JWMJ01000001
	Dickeya solani RNS07.7.3B	NZ_JWLR01000001
	Dickeya solani RNS 08.23.3.1.A	NZ_CP016928
	Dickeya solani RNS10-27-2A	NZ_VZQJ01000001
	Dickeya solani Sp1a	NZ_VZQL01000001
